# Loss or duplication of key regulatory genes coincides with environmental adaptation of the stomatal complex in *Nymphaea colorata* and *Kalanchoe laxiflora*

**DOI:** 10.1038/s41438-018-0048-8

**Published:** 2018-08-01

**Authors:** Meizhi Xu, Fei Chen, Shilian Qi, Liangsheng Zhang, Shuang Wu

**Affiliations:** 1 College of Horticulture, FAFU-UCR Joint Center and Fujian Provincial Key Laboratory of Haixia Applied Plant Systems Biology, Fujian Agriculture and Forestry University, Fuzhou, China; 2 State Key Laboratory of Ecological Pest Control for Fujian and Taiwan Crops; Key Laboratory of Ministry of Education for Genetics, Breeding and Multiple Utilization of Crops, Fujian Agriculture and Forestry University, Fuzhou, China

## Abstract

The stomatal complex is critical for gas and water exchange between plants and the atmosphere. Originating over 400 million years ago, the structure of the stomata has evolved to facilitate the adaptation of plants to various environments. Although the molecular mechanism of stomatal development in *Arabidopsis* has been widely studied, the evolution of stomatal structure and its molecular regulators in different species remains to be answered. In this study, we examined stomatal development and the orthologues of *Arabidopsis* stomatal genes in a basal angiosperm plant, *Nymphaea colorata*, and a member of the eudicot CAM family, *Kalanchoe laxiflora*, which represent the adaptation to aquatic and drought environments, respectively. Our results showed that despite the conservation of core stomatal regulators, a number of critical genes were lost in the *N. colorata* genome, including EPF2, MPK6, and AP2C3 and the polarity regulators BASL and POLAR. Interestingly, this is coincident with the loss of asymmetric divisions during the stomatal development of *N. colorata*. In addition, we found that the guard cell in *K. laxiflora* is surrounded by three or four small subsidiary cells in adaxial leaf surfaces. This type of stomatal complex is formed via repeated asymmetric cell divisions and cell state transitions. This may result from the doubled or quadrupled key genes controlling stomatal development in *K. laxiflora*. Our results show that loss or duplication of key regulatory genes is associated with environmental adaptation of the stomatal complex.

## How plants breathe in different environments

Researchers in China have shown how plants adapted to different environments by altering the structure of pores used for gas and water exchange, known as stomata. Shang Wu of Fujian Agriculture and Forestry University searched for stomatal development genes in the genomes of a range of plants, including a drought-tolerant species and a water lily, which diverged from other flowering plants early in their evolution. While the function of most stomatal genes has been conserved, some genes were absent in the water lily and some were doubled in the drought-resistant species. Analysis of the morphology of developing stomata in the two species revealed changes in the number and organization of cell divisions. Overall, the findings improve our understanding of how the evolution of stomata and may help guide the breeding or engineering improved varieties.

## Introduction

Stomata are a pore-like structure in multiple organs, including leaves and stems, which facilitates gas and water exchange. When environmental conditions are unfavourable, plants can regulate water evapotranspiration and reduce CO_2_ uptake by opening and closing the stomata. For instance, Crassulacean acid metabolism (CAM) plants are adapted to arid conditions^1^. The stomata in CAM plants remain closed during the day to reduce evapotranspiration while staying open at night to absorb CO_2_. These physiological traits make CAM plants resistant to diverse stresses, including strong irradiance and drought^2^.

Stomatal structure is highly conserved across land plants. The basic core structure with two guard cells surrounding the stomatal pore has remained unchanged during evolution^3^. However, the patterning of the mature stomatal structure differs among plant groups and can be generally summarized by three classes: anomocytic, stephanocytic, and paracytic^4^. The widely used model plant *Arabidopsis thaliana* exhibits anomocytic stomata. However, there are a few species (for example, CAM families) among the eudicots with paracytic stomata^5^. Most grass species have paracytic mature stomata^6^. *Amborella trichopoda* in ANITA possesses stephanocytic stomata^7^. The diverse architecture of mature stomatal structures may suggest the evolution of their different developmental regulations and their adaption to different environments.

In *A. thaliana*, meristem mother cells (MMCs) undergo up to three asymmetrical divisions to form guard mother cells (GMCs). In grasses, meristemoids divide asymmetrically to form GMCs, and the lateral neighbouring axial cell lineage surrounding the GMC undergoes asymmetric division to give rise to lateral subsidiary cells (LSCs)^8^. In *A. trichopoda*, however, protodermal cells can directly become GMCs or divide asymmetrically to produce a GMC^9^. Hence, the regulation of stomatal development is highly diverse in different groups of land plants.

In the past, *A. thaliana* and *Oryza sativa* were often used as model systems to study stomatal patterning and development. Based on those studies, we now have a good understanding of the basic molecular network behind stomatal development. In *A. thaliana*, a complex signalling cascade of several genes has been identified to promote stomatal development. The secreted peptides of the EPIDERMAL PATTERNING FACTOR (EPF)/EPF-LIKE (EPFL) family act with a mitogen-activated protein kinase (MAPK) cascade to regulate the activity of basic-helix-loop-helix (bHLH) transcription factors^10^. EPF1 and EPF2 specifically bind to leucine-rich repeat receptor (LRR) kinase complexes that include members of TOO MANY MOUTHS receptor-like protein (TMM) and the ERECTA family (ER). EPF1 is expressed in late-stage meristemoids, GMCs and young guard cells, whereas EPF2 is expressed in early-stage protodermal cells^11,12^. In the downstream pathway, a number of mitogen-activated protein (MAP) kinases, including MAPKKK YODA, MPKK4/5, MPKK7/9, and MAPK MPK3/6, were found to transduce the signalling for stomatal development^13^. Five bHLH transcription factors positively regulate the stomatal–lineage transition and differentiation. For example, SPEECHLESS (SPCH), MUTE, and FAMA act sequentially to promote the cellular transition in a stage-specific manner. SPCH regulates asymmetric divisions in MMC and MUTE involved in GMC differentiation^14,15^. FAMA promotes the last step to form GCs^16^. Two additional bHLH proteins, SCREAM/ICE1 and SCREAM2, act redundantly to heterodimerize SPCH, MUTE, and FAMA to coordinate the regulation^17^.

Polarity information is critical in stomatal development and directs asymmetric cell division and possibly cell fate determination. In *A. thaliana*, two unique polarity proteins, POLAR LOCALIZATION DURING ASYMMETRIC DIVISION AND REDISTRIBUTION (POLAR) and BREAKING OF ASYMMETRY IN THE STOMATAL LINEAGE (BASL), show mostly overlapping localization during asymmetric stomatal divisions^18,19^. In the grass, the asymmetric division taking place in the lateral neighbouring cell to produce the subsidiary cell relies on two LRR receptor-like kinases, PANGLOSS1 (PAN1) and PAN2^20,21^. PAN proteins are located at the poles in SMCs at the site of contact with GMCs, which precedes the polar accumulation of small GTPases (ROPs) and F-actin^22^. Interestingly, recent observations in *Brachypodium distachyon* found that BdMUTE regulates subsidiary cells through cell-to-cell movement^23^. In contrast, the MUTE homologue in *A. thaliana* is immobile^23,24^.

Although stomata morphologies across land plants have been widely examined, questions on the early evolution of angiosperms and the adaptation of stomata to diverse environments remain to be answered. It is not clear how molecular regulation of stomatal development evolved and how that relates to the diverse stomata morphologies among the land plants. Immediately above the root node of angiosperm evolution is the ANITA grade (basal angiosperms), which includes *Amborella*, *Nymphaeales*, *Illiciaceae*, *Trimeniaceae* and *Austrobaileyaceae*^7^. In this study, we took advantage of the newly sequenced genome of *Nymphaea colorata* (not released yet), a typical base angiosperm, to examine stomata regulation in early angiosperm evolution.

The structure and function of stomata are important for environmental adaptation. In some species, stomata underwent radical modifications to facilitate habituation to a particular environment. A recent study indicated that *Z. marina* lost all the genes involved in stomatal differentiation, which is coincident with its marine habituation. *Nymphaea colorata* is also an aquatic plant, so it is interesting to know if its stomata-related genes also changed during evolution. By contrast, *Kalanchoe laxiflora*, a CAM species, has adapted to drought conditions and has evolved specialized stomata functions. To understand how the evolution of the molecular regulation of stomatal development is associated with environmental adaptation, we analysed stomatal morphologies and related regulatory cascades in both *Nymphaea colorata* and *K. laxiflora*. Our analysis showed that although generally conserved, loss or duplication of key genes could be associated with structural and physiological renovations required for individual adaptation of plants to local environments.

## Materials and methods

### Plant materials and growth condition

*A. thaliana* Columbia seeds were germinated and grown on 1/2 MS medium with 1% agar, 1% sucrose and 0.05% (wt/vol) morpholinoethansulfonic acid monohydrate (pH 5.7) under a 16/8-h light/dark cycle at 23 °C. Plants were imaged 3–4 days after planting. *O. sativa* and *K. laxiflora* were grown at 28 °C with a 16/8-h light/dark photoperiod. *N. colorata* were cultivated in water at 23 °C in the greenhouse. Leaves of *Spirodela polyrhiza* were collected in winter 2017 at the Fujian Agriculture and Forestry University.

### Methods

#### Microscopy and image processing

For Differential Interference Contrast (DIC) imaging, the protocol was modified slightly according to Raissig et al.^23,25^. Samples from the mid-regions of leaves were cut into small squares and cleared using a solution (ethanol: acetic acid glacial, in proportions 4:1 by volume) to remove chlorophyll; then, samples were subjected to a basic solution (a mixture of 7% NaOH in 60% ethanol). Finally, samples were washed briefly with 40% ethanol and mounted in water for visualization and microscopy analysis. Samples were examined using a Nikon ECLIPSE Ni-U microscope fitted with a Nikon DS-Ri 2 digital camera. Images were processed using ImageJ.

#### Phylogenetic analysis

We surveyed a number of genomes, such as *A. thaliana*, *K. laxiflora*, *Sorghum bicolor*, *O. sativa*, *Zea mays*, *Ananas comosus*, *S. polyrhiza*, and *A. trichopoda*, from Phytozome v12. *Nelumbo nucifera* and *Phalaenopsis equestris* were retrieved from ftp://ftp.ncbi.nih.gov/genomes/. *Ginkgo biloba* was found from GigaDB (http://gigadb.org/). *N. colorata* was recently sequenced by Liangsheng Zhang’s Lab in Fujian Agriculture and Forestry University, and sequences were available in the water lily genome database (eplant.org). To obtain probable orthologous genes, we performed BLASTp (protein query–proteins database) and tBLASTn (protein query–nucleic acid database) searches to selectively look for similar protein sequences from these genomes^26^. A MAFFT (Multiple Sequence Alignment program) was chosen to produce an alignment of all amino-acid sequences with a BLAST score of at least 60 against *A. thaliana*^27^. The phylogenetic tree was reconstructed using the maximum likelihood (ML) method in FastTree2^28^.

Protein domains were identified using the National Center for Biotechnology Information conserved domain search tool. PEST domains were identified using emboss.bioinformatics.nl/cgi-bin/emboss/epestfind.

## Results

### Loss of stomatal development genes in *N. colorata*

It was reported that different stomatal development patterns occur in plants of the ANITA grade. *A. trichopoda* possesses mostly perigenous and mesoperigenous stomata^9^. In this species, protodermal cells can directly become GMCs or divide asymmetrically to produce GMCs and stomatal lineage ground cells^9^. However, in *Nymphaea*, protodermal cells seemed to skip asymmetric divisions and directly gave rise to GMCs^7,9^. It is still to be determined whether asymmetric division is an ancestral stomata-forming step during evolution.

**Fig. 1:**
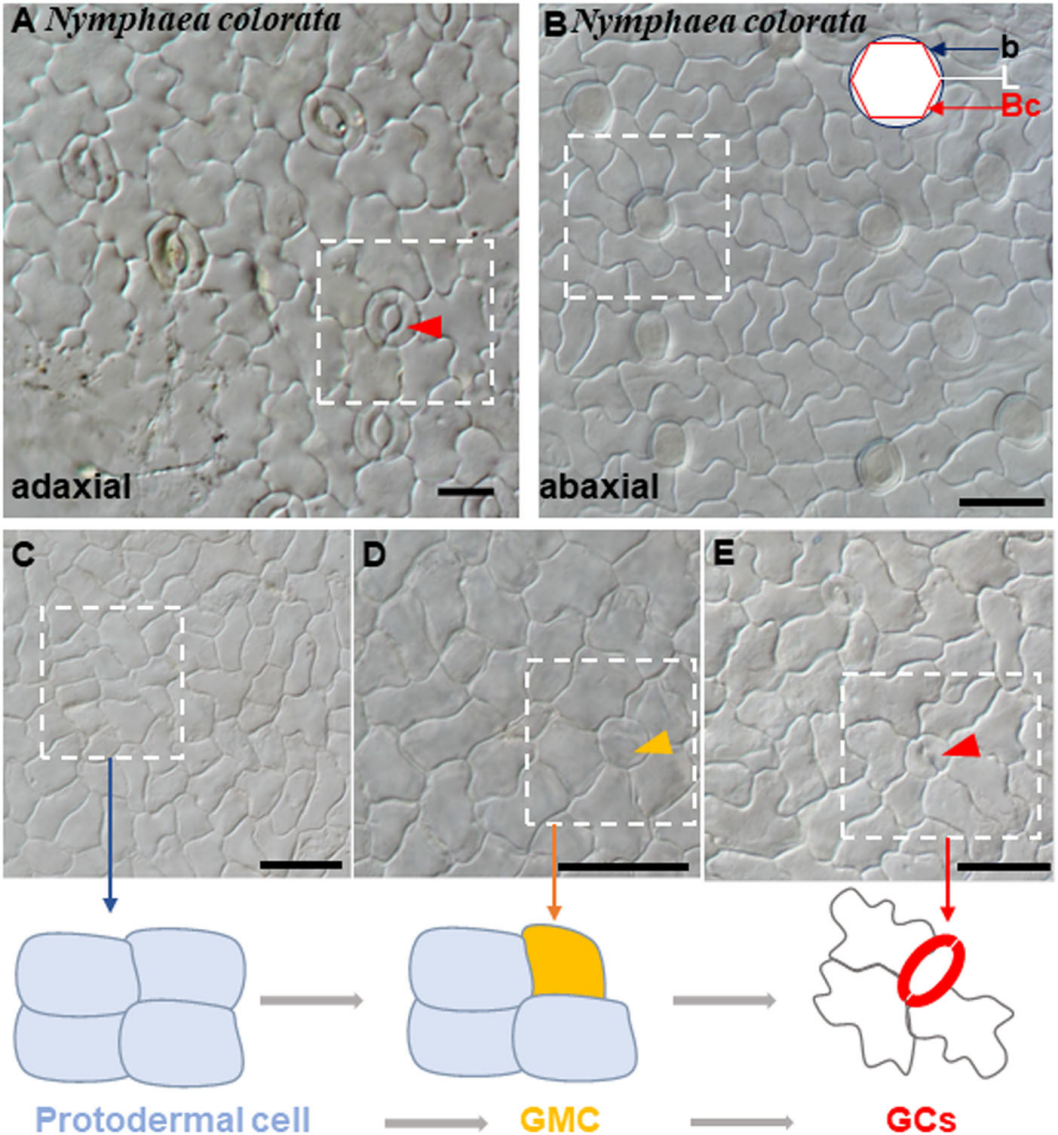
Stomatal structures and development process in *Nymphaea colorata*. **a** The upper epidermis of *N. colorata* with anomocytic stomata. **b** Abaxial hydropote complex structures of *N. colorata* with base (b) formed by anticlinal contact cell walls, the lens-shaped cell (L), and the bowl-shaped cell (Bc). **c-e** Micrograph of stomata at different developmental stages in adaxial leaf surfaces. **c** Squared patterning, a protodermal cell. **d** Large round cells are putative GMCs (orange arrow). **e** Stage with maturing stomata (red arrow). Schematic diagram of stomatal development. A protodermal cell (pale blue) that differentiated directly into a guard mother cell (orange); then, the GMC divided into GCs (red)

To gain a deeper understanding of the ancestral development of stomatal structure, we performed anatomic observation of the stomatal structure in *N. colorata*. We found that *N. colorata* stomata are only present on the adaxial surface of the floating leaf, with each stoma surrounded by 4–8 neighbouring cells (Fig. 1a). On the abaxial surface of *N. colorata*, we only found hydropote complexes with lens-shaped cells and bowl-shaped cells, which appeared to be surrounded by specialized rosettes of epidermal cells (Fig. 1b). It was hypothesized that the hydropote in *Nymphaea colorata* is homologous to stomatal complexes, and its functions and morphologies are highly associated with aquatic habitats^29^. Similarly, another floating plant, *S. polyrhiza*, has lost stomata on the abaxial surface (Figure S1). These results reveal that floating plants tend to lose stomata or create special stomata-like structures to adapt to the aquatic environment. It can also be exemplified by seagrass, *Zostera marina*, in which no stomata are present on leaves, and coincidently, entire stomatal genes are lost to adapt to the marine lifestyle^30^. Although anatomical descriptions of stomatal development have been reported for many taxa, little is known about the evolution of the molecular machine of stomatal formation across land plants.

**Fig. 2:**
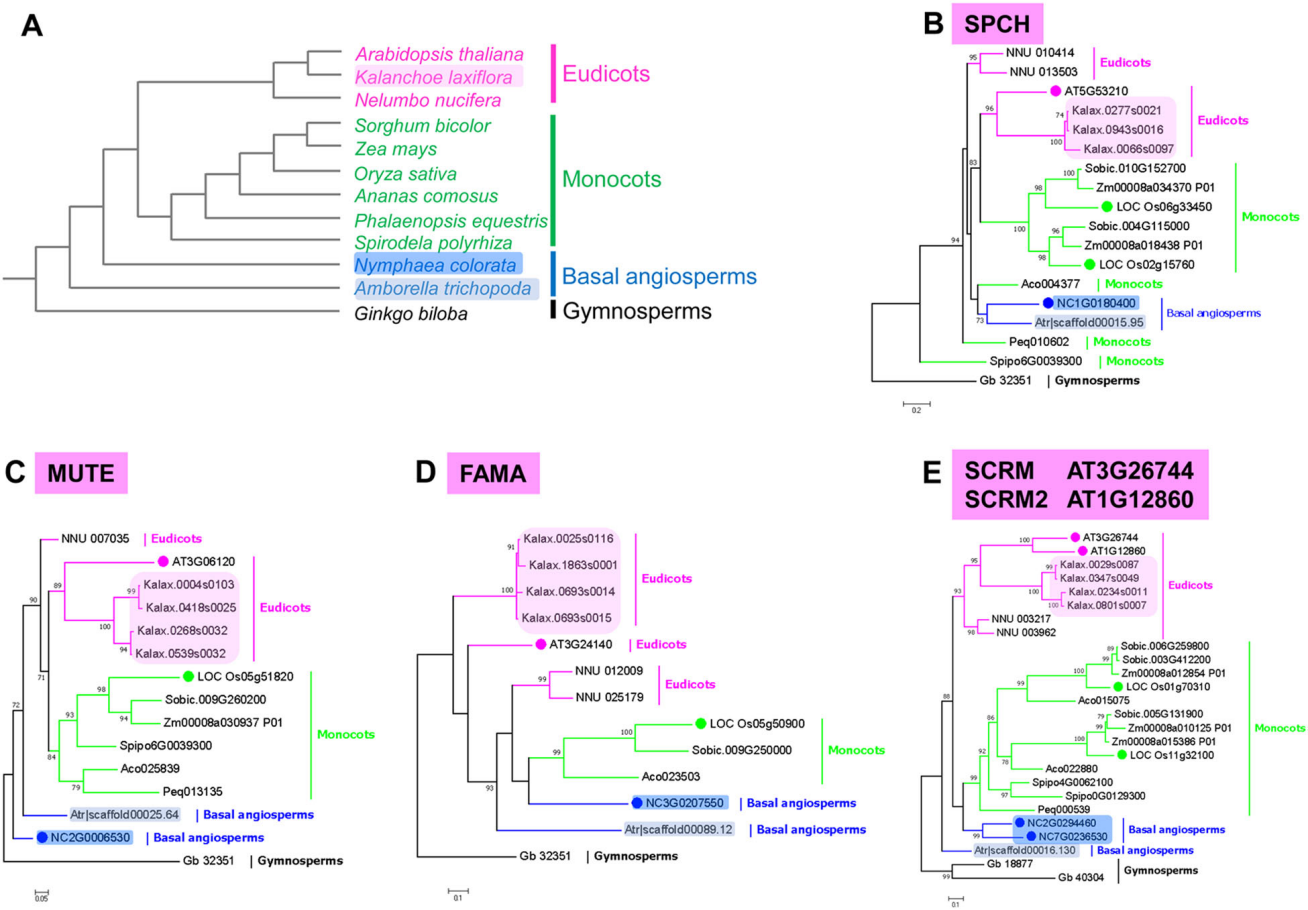
Phylogenetic trees of stomatal bHLH genes in representative species. **a** The molecular tree summarizes the phylogenetic relationships of representative species, including gymnosperms (e.g., *Ginkgo biloba*), basal angiosperms (e.g., *Amborella trichopoda* and *Nymphaea colorata*), monocots (e.g., *Oryza sativa* and *Spirodela polyrhiza*), and eudicots (e.g., *Arabidopsis thaliana* and *Kalanchoe laxiflora*). **b-e** Gene trees of master regulatory bHLH transcription factors SPCH (**b**), MUTE (**c**), FAMA (**d**) and ICE1/2 (**e**) in stomatal development. Amino-acid sequences from *G. biloba* (Gb), *A. trichopoda* (Atr, grey shade), *N. colorata* (Nc, blue shade), *S. polyrhiza* (Spipo), *Phalaenopsis equestris* (Peq), *Zea mays* (Zm), *O. sativa* (Loc_Os, green circle), *Nelumbo nucifera* (NNU), *K. laxiflora* (Kalax, peachy shade) and *A. thaliana* (AT, peachy circle) were used to generate trees

**Fig. 3:**
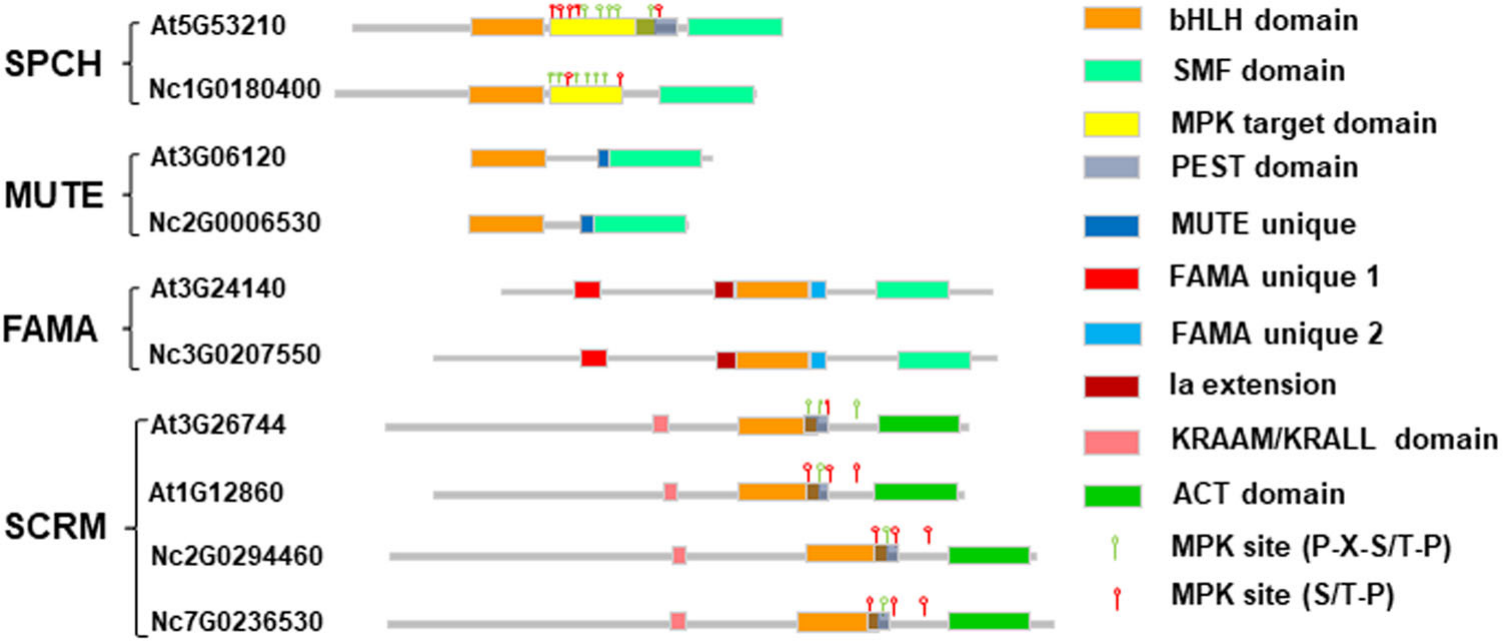
Schematics of the domain architecture of SPCH, MUTE, FAMA, and ICE-like sequences from *N. colorata* (Nc) and *A. thaliana* (At). NcSPCH shares the bHLH domain (orange) and C-terminal SMF domain (light blue) with AtSPCH but has no protein degradation-associated PEST domain (grey) and has a shorter MAPK target domain (yellow). Both NcMUTE and AtMUTE genes have a unique conserved region (MUTE unique, dark blue) and lack some residues preceding the bHLH domain that are present in all the other bHLH Ia members with various lengths. Both NcFAMA and AtFAMA genes have high AA sequence similarity and harbour three unique domains (FAMA unique 1, red; FAMA unique 2, blue; Ia extension, brown). Both NcICE-like and AtICE1/2 have highly conserved bHLH domains, potential PEST domains and ACT domains (green)

**Fig. 4:**
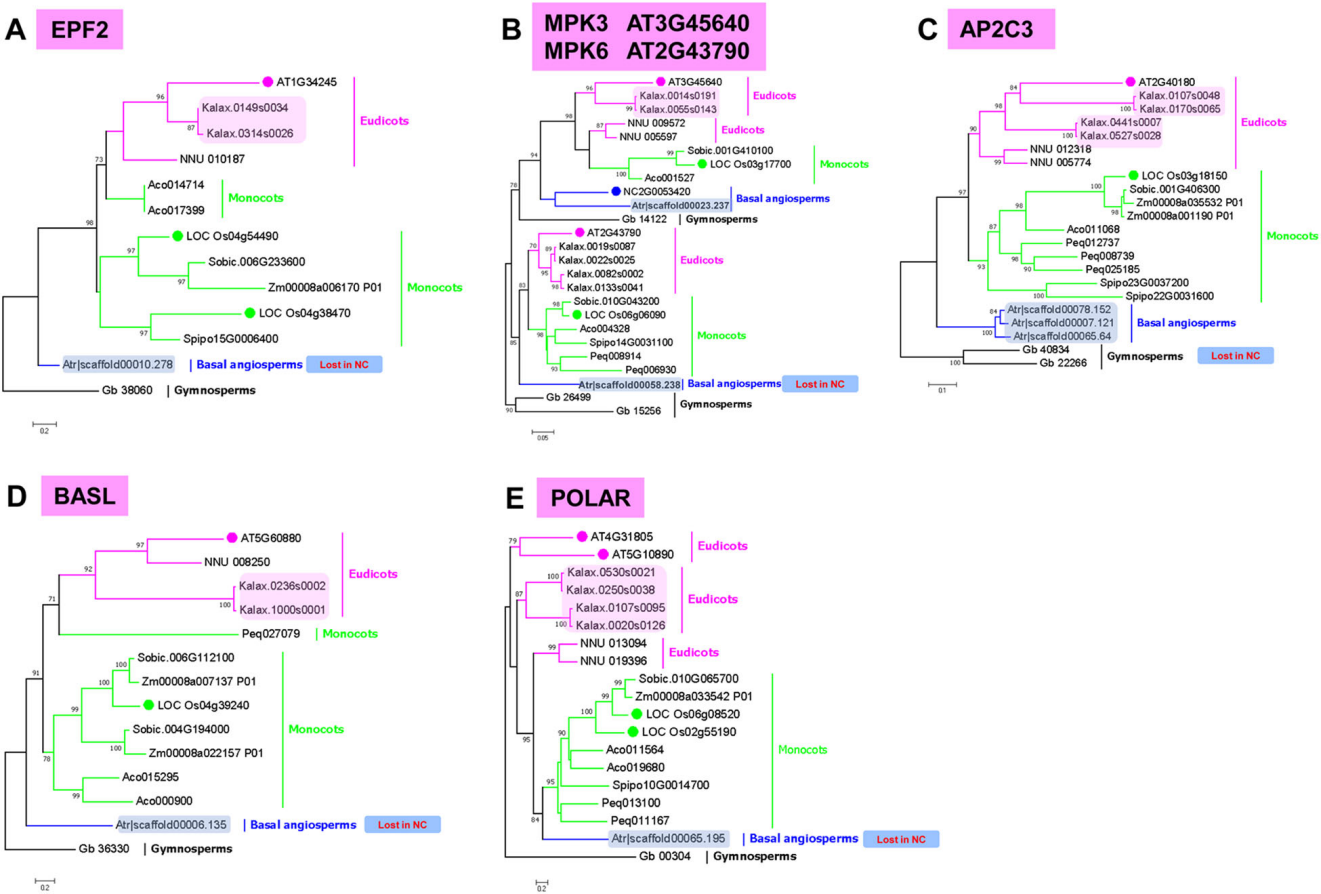
Phylogenetic analysis of genes lost in *N. colorata*. Phylogenetic trees constructed using amino-acid sequences of selected *A. thaliana* EPF2 (**a**), MPK3/MPK6 (**b**), AP2C3 (**c**), BASL (**d**) and POLAR (**e**) gene family members. Amino-acid sequences from *G. biloba* (Gb), *A. trichopoda* (Atr, grey shade), *N. colorata* (Nc, blue shade), *S. polyrhiza* (Spipo), *P. equestris* (Peq), *Z. mays* (Zm), *O. sativa* (Os, green circle), *N. nucifera* (NNU), *K. laxiflora* (Kalax, peachy shade) and *A. thaliana* (AT, peachy circle) were used to generate trees

One way to understand the evolution of these essential regulators of stomatal development is to analyse their phylogenies. This is currently feasible based on the genome sequences for many species, including the eudicots *A. thaliana* and *K. laxiflora*; the monocot plants *O. sativa* and *Z. mays*. To facilitate our understanding of the early evolution of these regulators, we included basal angiosperms *A. trichopoda*, and we recently sequenced the genome of an early-divergent angiosperm *N. colorata* (see Materials and methods for information on genome data) (Fig. 2a). To understand some special features of stomata formation in *N. colorata*, we analysed the potential orthologues of *A. thaliana* genes involved in stomatal formation using the unique unpublished genome data of water lily. In line with *A. thaliana*, we found high conservation of the core genes required for stomatal formation in *N. colorata*, including an orthologue of an SPCH-like gene, NcSPCH (Fig. 2b); orthologue of a MUTE-like gene, NcMUTE (Fig. 2c); orthologue of a FAMA-like gene, NcFAMA (Fig. 2d), and two orthologues of an ICE/SCRM-like gene, NcICE1 and NcSCRM2 (Fig. 2e). We further analysed the conservation of the homologous domain of these proteins and found a high degree of domain conservation (Fig. 3). However, we also found a number of genes missing from the *N. colorata* genome, including the peptide ligands EPF2, MPK6, and AP2C3 and the polarity controllers BASL and POLAR (Fig. 4). Interestingly, the function of lost genes seems to be highly specific to the asymmetric stomatal development stages.

### Stomatal development gene duplications in *K. laxiflora*

Whole-genome duplications (WGDs) are a common phenomenon during evolution, and the resulting gene duplications (GDs) provide redundant functions or specified novel functions^31–34^. WGDs are the source of functional diversity or novelty in the genome for adaption to environmental changes^35^. It has been suggested that two distinct WGDs occur in the *K. laxiflora* lineage and generate four gene copies across the genome^36^.

**Fig. 5:**
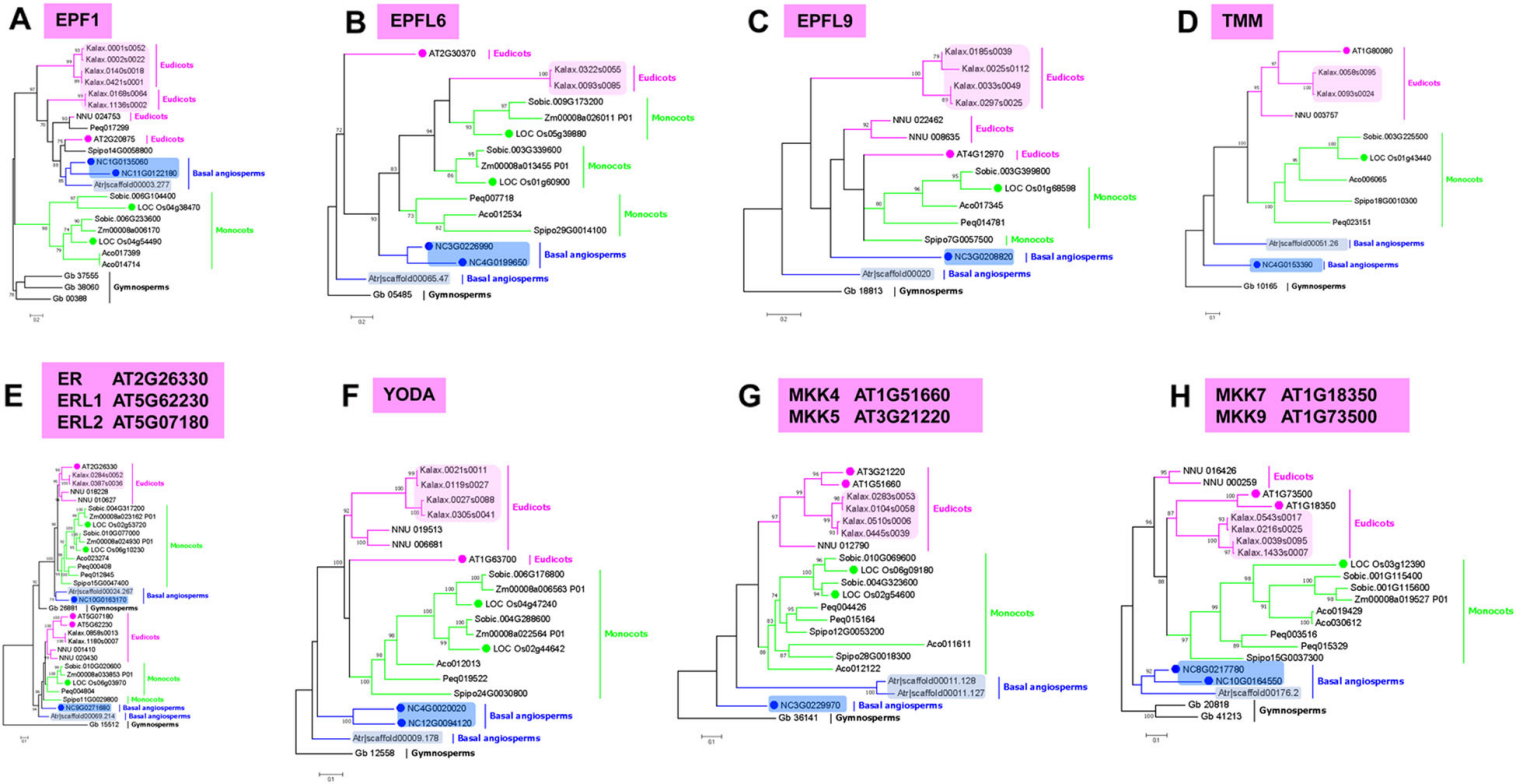
Phylogenetic analysis of stomatal regulators. **a-e** Phylogenetic analysis of ligand-receptor EPF/TMM/ER models. Phylogenetic trees constructed using amino-acid sequences of selected *A. thaliana* EPF1 (**a**), EPFL6 (**b**), EPFL9 (**c**), TMM (**d**) and ER/ERL (**e**) gene family members. **f-h** Phylogenetic analysis of the MAPK modules. Phylogenetic trees constructed using amino acid sequences of selected *A. thaliana* YODA (**f**), MKK4/5 (**g**), and MKK7/9 (**h**) gene family members. Amino-acid sequences from *G. biloba* (Gb), *A. trichopoda* (Atr, grey shade), *N. colorata* (Nc, blue shade), *S. polyrhiza* (Spipo), *P. equestris* (Peq), *Z. mays* (Zm), *O. sativa* (Os, green circle), *N. nucifera* (NNU), *K. laxiflora* (Kalax, peachy shade) and *A. thaliana* (AT, peachy circle) were used to generate trees

To understand the evolution of CAM stomata-related genes, we performed genome phylogenetic analysis in *K. laxiflora*. Intercellular signalling networks, such as peptide ligands, transmembrane receptors TMM/ER, MAPK modules, and bHLH transcription factors, are important for stomatal patterning^11–17^. In the EPF/TMM/ER module, our phylogenetic analysis shows that EPF2, EPFL6, TMM and ER/ERL have two copies, whereas EPF1 and EPFL9 have six and four orthologous genes, respectively, in *K. laxiflora* (Figs. 4a, 5). Furthermore we found each YODA, MKK4/MKK5, MKK7/MKK9, MAPKs MPK3/MPK6, and AP2C3 gene has only one copy in *A. thaliana* while expanded to four homologous genes in *K. laxiflora* (Figs. 4b, c, 5). Similarly, the group of bHLH transcription factors in *K. laxiflora* has also expanded to four orthologous (Fig. 2). In addition, the copy of the cell fate determining regulators, HDG2 and FLP/MYB88 also became quadrupled in *K. laxiflora* (Figures S2A, B). To understand if the asymmetric division is also associated with polarity in *K. laxiflora*, we analysed polar genes in *K. laxiflora*. Our analysis indicates that *K. laxiflora* genome contains homologous genes for PAN1, PAN2, POLAR, BASL, and ROP (Figures S2C, S2D, S2E; Fig. 4d, e). Together, these findings suggest that four copies of stomatal orthologous genes in *K. laxiflora* possibly derived from maximally two rounds of genome duplication (Table 1).

**Table 1:** Gene involved in stomata development in *N. colorata* compared with other representative plant

Gene name	Symbol	*A. thaliana*	*K. laxiflora*	*N. nucifera*	*O. sativa*	*S. polyrhiza*	*N. colorata*	*A. trichopoda*	*G. biloba*
Differentiation genes									
SPEECHLESS	SPCH	AT5G53210	Kalax.0066s0097 Kalax.0943s0016 Kalax.0277s0021	NNU 010414 NNU 013503	LOC Os06g33450 LOC Os02g15760	Spipo6G0039300	NC1G0180400	Atr|scaffold00015.95	Gb 32351
MUTE	MUTE	AT3G06120	Kalax.0004s0103 Kalax.0418s0025 Kalax.0268s0032 Kalax.0539s0032	NNU 007035	LOC Os05g51820		NC2G0006530	Atr|scaffold00025.64	
FAMA	FAMA	AT3G24140	Kalax.0693s0014 Kalax.1863s0001 Kalax.0693s0015 Kalax.0693s0014	NNU 012009 NNU 025179	LOC Os05g50900	NF	NC3G0207550	Atr|scaffold00089.12	
SCREAM/ICE1	SCRM	AT3G26744	Kalax.0347s0049 Kalax.0029s0087 Kalax.0801s0007 Kalax.0234s0011	NNU 003962 NNU 003217	LOC Os11g32100 LOC Os01g70310	Spipo4G0062100 Spipo0G0129300	NC2G0294460 NC7G0236530	Atr|scaffold00016.130	Gb 18877Gb 40304
SCREAM2	SCRM2	AT1G12860							
FOUR LIPS	FLP	AT1G14350	Kalax.0757s0004 Kalax.0556s0006 Kalax.0031s0030 Kalax.0089s0020	NNU 022886 NNU 000781	LOC Os07g43420	Spipo0G0157900	NC2G0034590	Atr|scaffold00010.370	Gb 06045
MYB88	MYB88	AT2G02820							
HOMEODOMAIN GLABROUS2	HDG2	AT1G05230	Kalax.0393s0043 Kalax.0069s0102 Kalax.1016s0007 Kalax.1527s0001	NNU 019425 NNU 014296	LOC Os04g53540 LOC Os08g08820 LOC Os08g04190	Spipo7G0015400	NC1G0306950	Atr|scaffold00004.265	Gb 18862Gb 16030
Spacing and patterning genes									
EPIDERMAL PATTERNING FACTOR1	EPF1	AT2G20875	Kalax.0168s0064 Kalax.1136s0002 Kalax.0140s0018 Kalax.0421s0001 Kalax.0001s0052 Kalax.0002s0022	NNU 024753	LOC Os04g54490 LOC Os04g38470	Spipo14G0058800	NC1G0135060 NC11G0122180	Atr|scaffold00003.277	Gb 37555Gb 00388Gb 38060
EPIDERMAL PATTERNING FACTOR2	EPF2	AT1G34245	Kalax.0149s0034 Kalax.0314s0026	NNU 010187		Spipo15G0006400	NF	Atr|scaffold00010.278	
STOMAGEN/EPF-LIKE9	EPFL9	AT4G12970	Kalax.0185s0039 Kalax.0025s0112 Kalax.0033s0049 Kalax.0297s0025	NNU 022462 NNU 008635	LOC Os01g68598	Spipo7G0057500	NC3G0208820	Atr|scaffold00020	Gb 18813
CHALLAH/EPF-LIKE6	EPFL6	AT2G30370	Kalax.0093s0085 Kalax.0322s0055	NF	LOC Os01g60900 LOC Os05g39880	Spipo29G0014100	NC3G0226990 NC4G0199650	Atr|scaffold00065.47	Gb 05485
ERECTA	ER	AT2G26330	Kalax.0387s0036 Kalax.0284s0052	NNU 018228 NNU 010627	LOC Os06g10230 LOC Os02g53720	Spipo15G0047400	NC10G0163170	Atr|scaffold00024.267	Gb 26881
ERECTA-LIKE1	ERL1	AT5G62230	Kalax.0858s0013 Kalax.1180s0007	NNU 001410	LOC Os06g03970	Spipo11G0029800	NC9G0271680	Atr|scaffold00069.214	Gb 15512
ERECTA-LIKE2	ERL2	AT5G07180		NNU 020430					
TOO MANY MOUTHS	TMM	AT1G80080	Kalax.0093s0024 Kalax.0058s0095	NNU 003757	LOC Os01g43440	Spipo18G0010300	NC4G0153390	Atr|scaffold00051.26	Gb 10165
STOMATAL DENSITY AND DISTRIBUTION1	SDD1	AT1G04110	Kalax.0525s0015 Kalax.0155s0004	NNU 010999	LOC Os03g04950	Spipo1G0013100	NC4G0239300	Atr|scaffold00039.113	Gb 35657
CO2 RESPONSE SECRETED PROTEASE	CRSP	AT1G20160	NF	NNU 013210	LOC Os09g30458	Spipo3G0019800	NC2G037260	Atr|scaffold00152.21	Gb 39463
YODA	YDA	AT1G63700	Kalax.0027s0088 Kalax.0305s0041 Kalax.0021s0011 Kalax.0119s0027	NNU 019513 NNU 006681	LOC Os02g44642 LOC Os04g47240	Spipo24G0030800	NC4G0020020 NC12G0094120	Atr|scaffold00009.178	Gb 12558
MPK3	MPK3	AT3G45640	Kalax.0014s0191 Kalax.0055s0143	NNU 009572 NNU 005597	LOC Os03g17700	NF	NC2G0053420	Atr|scaffold00023.237	Gb 14122
MPK6	MPK6	AT2G43790	Kalax.0019s0087 Kalax.0022s0025 Kalax.0082s0002 Kalax.0133s0041	NF	LOC Os06g06090	Spipo14G0031100	NF	Atr|scaffold00058.238	Gb 26499Gb 15256
MKK4	MKK4	AT1G51660	Kalax.0510s0006 Kalax.0445s0039 Kalax.0283s0053 Kalax.0104s0058	NNU 012790	LOC Os02g54600 LOC Os06g09180	Spipo12G0053200 Spipo28G0018300	NC3G0229970	Atr|scaffold00011.127 Atr|scaffold00011.128	Gb 36141
MKK5	MKK5	AT3G21220							
MKK7	MKK7	AT1G18350	Kalax.0543s0017 Kalax.0216s0025 Kalax.0039s0095 Kalax.1433s0007	NNU 016426 NNU 000259	LOC Os03g12390	Spipo15G0037300	NC8G0217780 NC10G0164550	Atr|scaffold00176.2	Gb 41213Gb 20818
MKK9	MKK9	AT1G73500							
ARABIDOPSIS PROTEIN PHOPHATASE 2C	AP2C3	AT2G40180	Kalax.0107s0048 Kalax.0170s0065 Kalax.0441s0007 Kalax.0527s0028	NNU 012318 NNU 005774	LOC Os03g18150	Spipo22G0031600 Spipo23G0037200	NF	Atr|scaffold00065.64 Atr|scaffold00078.152 Atr|scaffold00007.121	Gb 40834Gb 22266
Polarity and division asymmetry genes									
PANGLOSS1	PAN1	AT2G42290, AT3G57830	Kalax.0222s0039 Kalax.0637s0020	NNU 012890	LOC Os08g39590	Spipo12G0035200	NC1G0088630	Atr|scaffold00022.305	Gb 28844
PANGLOSS2	PAN2	AT4G20940	Kalax.0016s0247 Kalax.0114s0005	NNU 026348	LOC Os07g05190	Spipo32G0009300 Spipo0G0142000	NC14G0281210	Atr|scaffold00175.33	Gb 30406Gb 18587
RHO-RELATED PROTEIN FROM PLANTS 9	ROP9	AT4G28950	Kalax.0192s0051 Kalax.0015s0042 Kalax.1214s0006	NNU 005916 NNU 003451	LOC Os05g43820	Spipo26G0003200	NC6G0252910	Atr|scaffold00002.129	Gb 09833
BREAKING OF ASYMMETRY IN THE STOMATAL LINEGAE	BASL	At5g60880	Kalax.0236s0002 Kalax.1000s0001	NNU 008250	LOC Os04g39240	NF	NF	Atr|scaffold00006.135	Gb 36330
POLAR LOCALIZATION DURING ASYMMETRIC DIVISION AND REDISTRIBUTION	POLAR	AT4G31805	Kalax.0020s0126 Kalax.0107s0095 Kalax.0250s0038 Kalax.0530s0021	NNU 019396NNU 013094	LOC Os06g08520 LOC Os02g55190	Spipo10G0014700	NF	Atr|scaffold00065.195	Gb 00304
Mitosis and cytokinesis genes									
STOMATAL CYTOKINESIS DEFECTIVE 1	SCD1	AT1G49040	Kalax.0061s0068 Kalax.0190s0062	NNU 012674	LOC Os01g39380	Spipo21G0025200	NC3G0202830	Atr|scaffold00104.16	Gb 36258
Hormone and environmental signalling genes									
CRYPTOCHROME	CRY1	AT4G08920	Kalax.0428s0010 Kalax.1365s0004 Kalax.0290s0014 Kalax.0239s0053	NNU 001876 NNU 015266	LOC Os04g37920 LOC Os02g36380	Spipo15G0011900	NC8G0218290	Atr|scaffold00038.124	Gb 13122
	CRY2	AT1G04400	Kalax.0094s0015 Kalax.0075s0050	NNU 010890 NNU 018834	LOC Os02g41550	Spipo1G0003600	NC12G0249420	Atr|scaffold00148.69	Gb 13122
PHYTOCHROME	PHYA	AT1G09570	Kalax.0106s0002 Kalax.0005s0079 Kalax.0038s0184 Kalax.0172s0035	NNU 026354	LOC Os03g51030	Spipo6G0014200	NC10G0166490	Atr|scaffold00045.165	Gb 21967
	PHYB	AT2G18790	Kalax.0613s0014 Kalax.0391s0019 Kalax.0996s0003	NNU 014452	LOC Os03g19590	Spipo6G0031800	NC5G0160900	Atr|scaffold00003.45	Gb 17897
PYTOCHROME-INTERACTING FACTOR 4	PIF4	AT2G43010	Kalax.0495s0020 Kalax.0759s0011	NNU 026428	LOC Os03g43810 LOC Os07g05010 LOC Os03g56950	Spipo13G0048400	NC10G0166270	Atr|scaffold00039.9	Gb 07156
CONSTITUTIVE PHOTOMORPHOGENIC 1	COP1	AT2G32950	Kalax.0049s0041 Kalax.0049s0041	NNU 005078 NNU 015709	LOC Os02g53140	Spipo31G0000500	NC1G0178350	Atr|scaffold00074.24	Gb 15627
CONSTITUTIVE PHOTOMORPHOGENIC 10	COP10	AT3G13550	Kalax.0340s0003 Kalax.0021s0072	NNU 019762	LOC Os07g38940	Spipo2G0063200	NC1G0193740	Atr|scaffold00061.43	Gb 07763
HIGH CARBON DIOXIDE	HIC1	AT2G46720	Kalax.0018s0006 Kalax.0090s0007 Kalax.1015s0012 Kalax.0013s0142 Kalax.1015s0014	NNU 006085 NNU 003630	LOC Os05g49900 LOC Os02g11070 LOC Os06g39750	Spipo14G0001700 Spipo21G0006400	NC6G0254440 NC1G0129310	Atr|scaffold00052.41	Gb 23820
BRI SUPPRESSOR1	BSU1	AT1G03445	Kalax.0084s0077 Kalax.1286s0001 Kalax.0045s0074 Kalax.0289s0053	NNU 001649 NNU 024344	LOC Os05g05240	Spipo6G0007500	NC1G0193170	Atr|scaffold00004.204	Gb 36990
BRASSINOSTEROID INSENSTIVIE 2	BIN2	AT4G18710	Kalax.0092s0006 Kalax.0164s0037 Kalax.1441s0002 Kalax.0375s0036 Kalax.0283s0042 Kalax.0104s0069	NNU 025519	LOC Os01g10840 LOC Os05g11730 LOC Os02g14130 LOC Os06g35530	Spipo18G0019800 Spipo14G0030500	NC9G0114290 NC13G0028550	Atr|scaffold00170.9	Gb 21469

*NF* not found

### Novel formation of subsidiary cells in *K. laxiflora*

CAM increases water-use efficiency and drought resistance in plants, which is characterized by nocturnal opening and diurnal closing of the stomata^36^. Therefore, stomatal control in the leaves is particularly important for this type of plant to reduce evapotranspiration in the daytime and increase carbon dioxide (CO_2_) collection at night^2,36^. The physiological traits probably improve the resistance of CAM plants to diverse environmental stresses, including drought^1,2^.

**Fig. 6:**
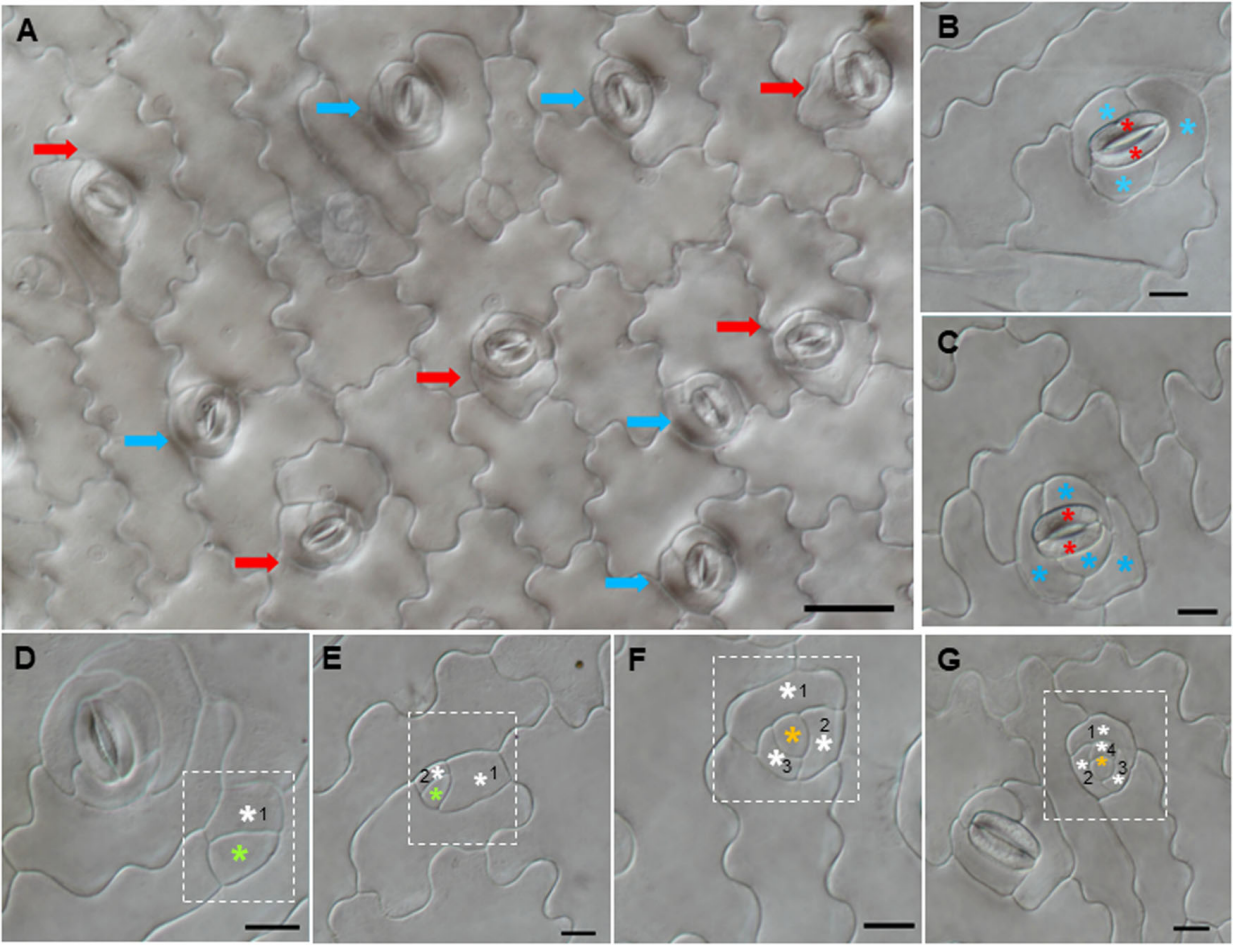
Stomatal development of *Kalanchoe laxiflora* on adaxial leaf epidermis. **a** There are two types of mature stomata equably distributed on adaxial leaf surfaces; the guard cells are surrounded by three (blue arrow) or four subsidiary cells (red arrow). **b** A stoma with three subsidiary cells. **c** A stoma with four subsidiary cells. **d-g** DIC of different stages with asymmetric division finally form two mature stomatal types. Meristemoid (green star), surrounding cells (white star), guard mother cell (orange star), guard cells (red star), and subsidiary cells (blue star)

To gain a better understanding of the stomatal complex in CAM plants, we performed anatomical observation of *K. laxiflora*, a member of the eudicot CAM family. In *K. laxiflora*, stomata are surrounded by three to four small subsidiary cells in adaxial leaf surfaces (Fig. 6a). Similarly, we found that the stomata of *Phalaenopsis equestris*, another CAM monocot species, is also surrounded by approximately four subsidiary cells (Figure S3). This innovation of stomatal architecture could derive from differential regulation of stomatal formation. We found that in *K. laxiflora*, stomata formed via a series of asymmetric cell divisions and cell state transitions: protodermal cells entered the stomatal lineage and took on a MMC identity; the MMC underwent three or four asymmetrical divisions to form GMC and Stomatal lineage ground cell (SLGC) (Fig. 6d-g). The GMC underwent a symmetric division to form a pair of guard cells, and SLGCs eventually became subsidiary cells surrounding the guard cell (Fig. 6b, c).

**Fig. 7:**
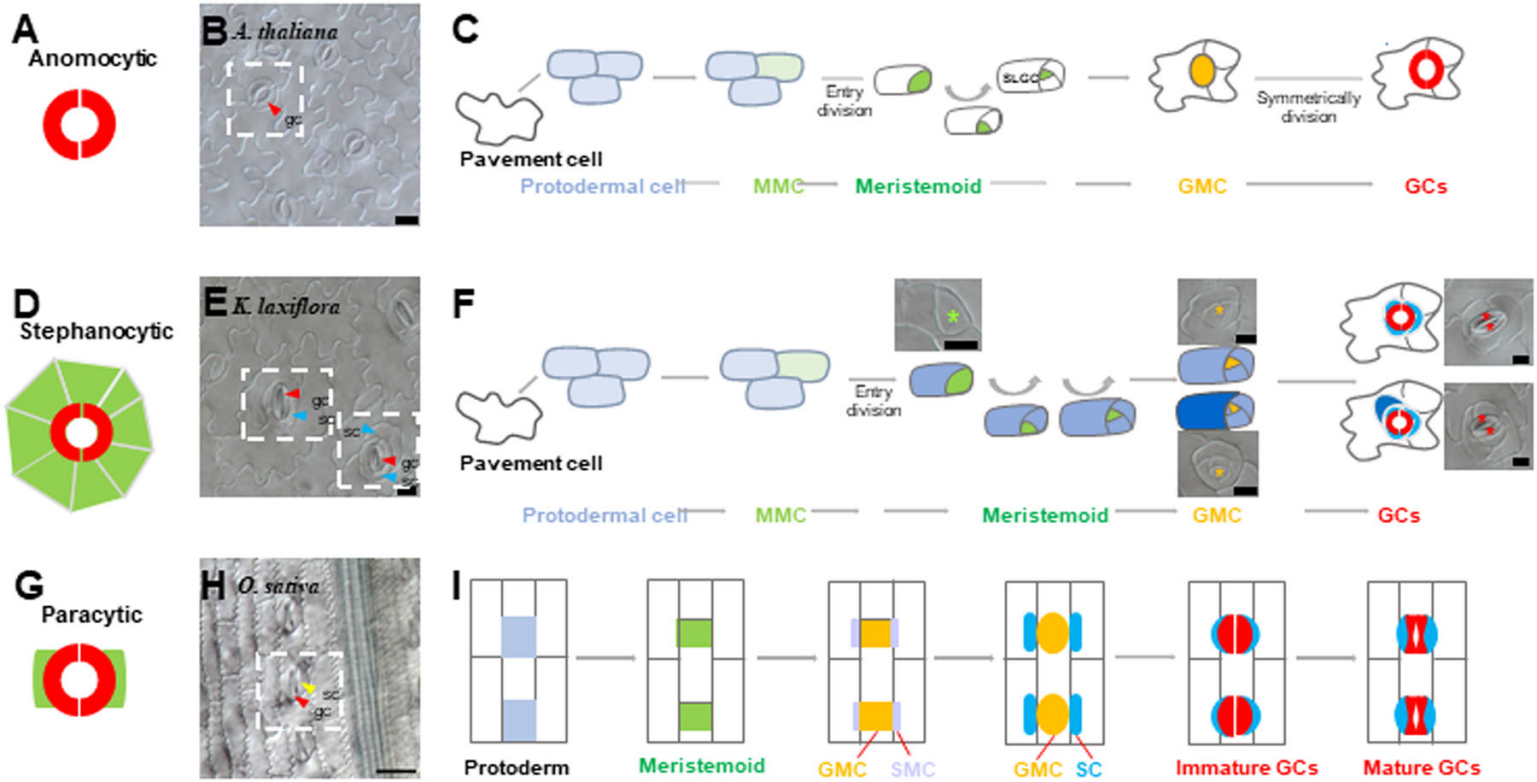
Mature stomatal types and development in diverse species. **a**, **d**, **g** Mature stomatal types. Diagrams show the guard cell pair (red) and subsidiary cells (green). **a** Anomocytic stomata lack subsidiary cells. **d** Stephanocytic stomata possess a ring of subsidiary cells. **g** Paracytic stomata possess one pair of lateral subsidiary cells oriented parallel to the guard cells. **b**, **c** Example of eudicot stomata in *A. thaliana*. **b** The upper epidermis of *A. thaliana* with anomocytic stomata. **c** Schematic diagram of stomatal development transitions. A subset of protodermal cells (pale blue) enter the stomatal lineage and take on an MMC identity; the MMC (pale green) undergoes asymmetric cell division producing a smaller meristemoid (green) and larger SLGCs (white). Then, the meristemoid differentiates into a GMC (orange), and the GMC undergoes a symmetric division to form a pair of guard cells (red). **e**, **f** Example of eudicot stomata in *K. laxiflora*. **e** The upper epidermis of *K. laxiflora* with stephanocytic stomata. **f** Schematic diagram of stomatal development. Protodermal cells (pale blue) take on an MMC identity. The MMC (pale green) divides through three or four asymmetric divisions to give rise to a GMC (orange), and a round of neighbouring cells (dark blue) eventually become subsidiary cells (blue) surrounding the guard cells (red). **h**, **i** Example of monocot stomata in *O. sativa*. **h** The upper epidermis of *O. sativa* with linear cell files and paracytic stomata. **i** Diagrams illustrating stomatal development for the stomatal complex. Cell protoderm files (pale blue) asymmetrically divide to create a meristemoid (green), and the meristemoid differentiates into a GMC (orange). Then, neighbouring cell files (SMC, pale purple) divide asymmetrically to form SCs (blue). Finally, the GMC divides once symmetrically to form GCs (red), and the GCs and SCs terminally differentiate and form mature dumbbell-shaped stomata. Key: protodermal cell that will give rise to the stomatal lineage, pale blue; MMC (meristemoid mother cell), pale green; meristemoid, green; SLGCs (stomatal-lineage ground cell), white; GMC (guard mother cell), orange; GCs (guard cells), red; SMC (subsidiary mother cell), pale purple; SCs (subsidiary cells), blue

**Fig. 8:**
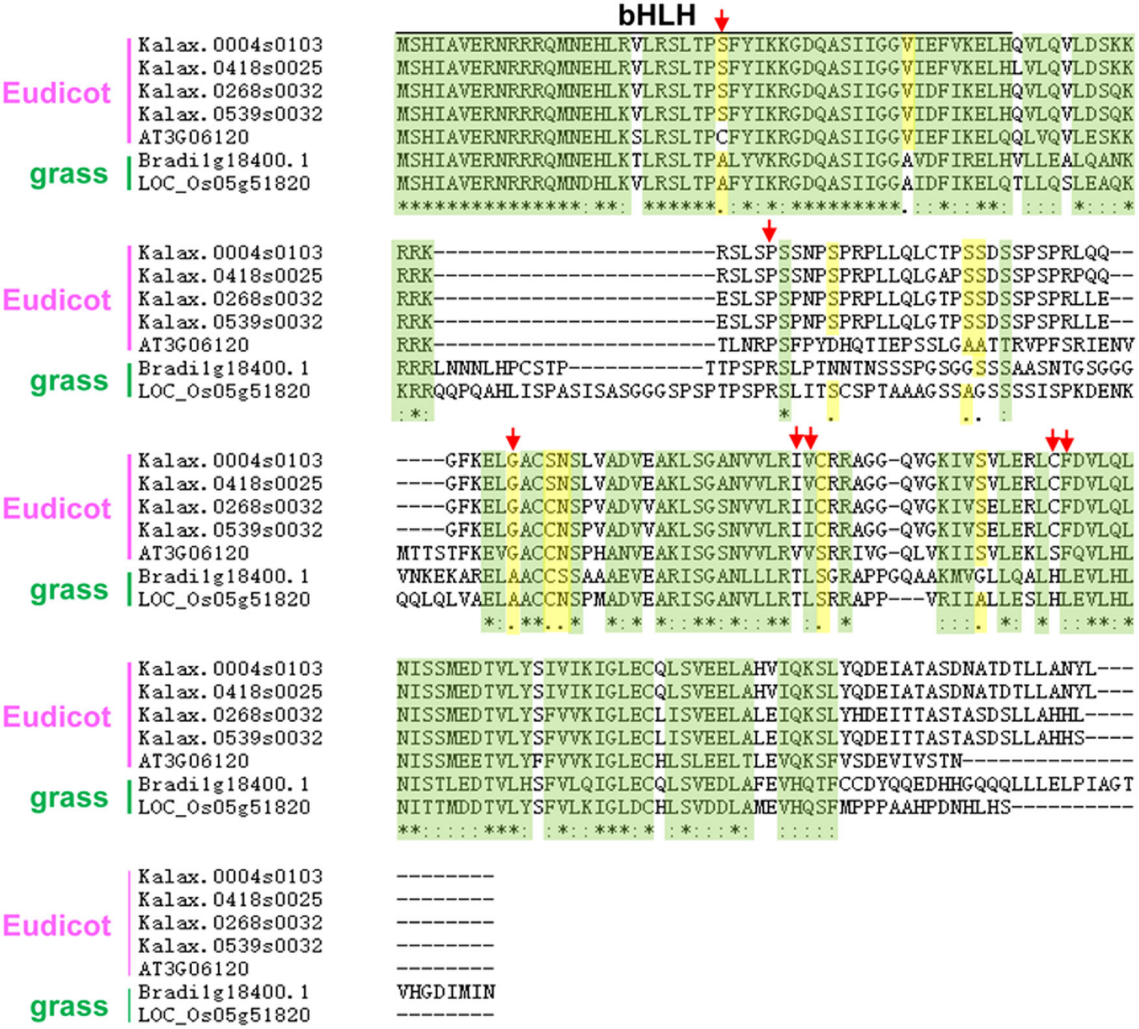
Alignment of grass and eudicot MUTE orthologues to identify potential mobility residues. MUTE orthologues of the representative grass species *Brachypodium* (BdMUTE—Bradi1g18400) and rice (OsMUTE—LOC_Os05g51820) were aligned with the MUTE orthologues of the representative eudicot species *Arabidopsis* (AtMUTE—AT3G06120) and Kalax.0004s0103/Kalax.0418s0025/Kalax.0268s0032/Kalax.0539s0032 using ClustalW (http://www.genome.jp/tools-bin/clustalw). The bHLH domain spans the first 50 amino acids and is indicated. Green shaded amino acids represent high similarity, whereas yellow shaded amino acids represent intermediate similarity. Candidate amino acids that are either consistently different between grasses and eudicots or are conserved among grasses but not in eudicots, or vice versa, are marked with a red asterisk and represent potential mobility motifs

It is widely accepted that different stomatal patternings reflect the asymmetric division of precursor cells and lateral divisions of neighbouring cells^37^. For example, in anomocytic stomata occurring in the eudicot *A. thaliana* (Fig. 7a, b), the MMC underwent three asymmetric divisions to give rise to a GMC and SLGCs, which was followed by a transition from SLGCs to pavement cells (Fig. 7c). Although both *A. thaliana* and *K. laxiflora* are eudicots, *K. laxiflora* possesses stephanocytic stomata (Fig. 7d, e). Developmentally, there is a similarity between these two types of stomata: meristemoids undergo a series of asymmetric divisions to produce SLGCs surrounding guard cells (Fig. 7f), and different cell fate choices of SLGCs finally give rise to different stomatal complexes (Figure S4). In monocot species such as *O. sativa*, the type of mature stomata is named the paracytic type, in which the guard cell is surrounded by two subsidiary cells (Fig. 7g, h). In this type, the stomatal meristemoid divides asymmetrically to form a larger SLGC and a smaller meristemoid that directly forms the GMC. Before the GMC divides, it induces neighbouring cell files to adopt an SMC identity, which subsequently forms SCs via asymmetric divisions. The GMC then undergoes symmetric mitosis to eventually form guard cells (Fig. 7i). Therefore, subsidiary cells can develop through different ways: one is through asymmetric division in *O. sativa*, and the other is through SLGC differentiation in *K. laxiflora*. In *K. laxiflora*, subsidiary cells are noticeably visible, but little is known about the factors defining subsidiary cell identity. In *Brachypodium distachyon*, subsidiary cells are formed through asymmetric divisions. BdMUTE is an orthologue of *A. thaliana* MUTE that has been identified as sufficient for SC formation based on its acquisition of cell-to-cell mobility^23^. In *A. thaliana*, AtMUTE, which is associated with GMC identity, is nonmobile. The question is whether the KalaxMUTE could also specify SC identity by being mobile. To address this, we compared MUTE orthologues of the representative species with *B. distachyon*, *A. thaliana* and *K. laxiflora* to test potential mobility motifs in *K. laxiflora* (Fig. 8). Our results show high conservation in the bHLH functional domain. The differences in potential mobility residues of KalaxMUTE from its homologue in *B. distachyon* are similar to those in *A. thaliana*. Thus, the subsidiary cells in *K. laxiflora* may not be specified by KalaxMUTE mobility.

## Discussion

Stomatal patterning is diverse among different land plants. In *Physcomitrella patens*, stomata exhibit partial or complete division to form a single GC or paired GCs, respectively^38^. Moss does not have genes encoding MUTE or SPCH and uses genes encoding two bHLH proteins, PpSMF1 and PpSCRM1, to promote stomatal formation^39^. In *A. thaliana*, the stomata are surrounded by two kidney-shaped guard cells, and polar localization of BASL is required for a series of asymmetric divisions to form the stomatal structure^40^. In *O. sativa*, polar localization of PAN protein is responsible for subsidiary cell asymmetry in the stomatal complex^10^. In *B. distachyon*, BdMUTE is necessary and sufficient for SC formation. However, AtMUTE in *A. thaliana* defines GC precursor fate^23^. Overall, it appears that the function of most genes is conserved during stomatal formation across plant evolution, but there are novel genes recruited to regulate unique aspects of stomatal patterning in some species.

The regulatory machine of stomata development appeared to be flexible and adaptable during evolution. The adaptation pressure could quickly change the division and differentiation pattern during stomata formation. For example, all the genes involved in stomatal differentiation are lost in seagrass Zostera to enhance its adaptation to marine lifestyle^30^. Plants of the ANITA grade form specialized structures in the epidermal cells to adapt to its habitat^29^. Similarly, *N. colorata* has lost genes, which could be associated with its unique stomatal development. However, further molecular and genetic manipulations are needed for functional verification.

Compared with our understanding of stomatal development in model systems, little is known about the molecular evolution of stomatal morphology, particularly in basal angiosperms. Alongside the completion of the genome, we are beginning to find the comparative molecular basis of the evolution of stomatal development and identify orthologues of stomatal regulator genes in a selected range of phylogenetic taxa. However, it is still technically difficult to analyse the function of orthologues. In the *N. colorata* genome, we found that a number of the genes that are highly specific to the stomatal asymmetric division were missing. Taken together, these results suggest that most core regulators of stomata formation remain conserved during evolution, whereas some gene loss events can occur to modify stomata formation processes, such as asymmetric division. These changes at the genetic and morphological levels of individual species may result from adaptation to inhabitant environments rather than evolutionary changes.

Recent studies have indicated that WGD events are ubiquitous in the evolution of angiosperms, and WGDs tend to retain multiple family duplications to increase the frequency of multiplication and the function of genes^41^. Thus, WGDs are widely thought to provide genomic novelties and complexities to promote plant adaptation to environments^42^. Large-scale GDs involved in stomata development through WGDs in *K. laxiflora* have been identified^36^.

Analysis of the genes involved in stomata formation showed that the protein sequences of the core genes required to instigate and pattern stomata are conserved in *K*. *laxiflora* (Table 1). It is unclear whether the expression or protein modification of these regulators is different in *K*. *laxiflora* compared with that in *A*. *thaliana*. Indeed, the duplication of stomata regulator genes appears to be a common theme in *K. laxiflora*, but the extent to which this represents a divergence in gene function requires further studies.

It seemed that genes encoding critical developmental regulators were more likely to be retained during evolution^43,44^. For stomatal development, subsidiary cells can occur from an adjacent cell file or the same cell as the guard cells. Based on sequence conservation, the mobility of KalaxMUTE could be similar to its homologue in *Arabidopsis*. Thus, it is less likely that the modification of KalaxMUTE leads to featured stomatal subsidiary cells in *K. laxiflora*. Further work is needed to investigate whether the gene gains in *K. laxiflora* are associated with subsidiary cell establishment.

## Electronic supplementary material

Supplemental Figure 1

Supplemental Figure 2

Supplemental Figure 3

Supplemental Figure 4
