# Supplementary figures and images for: Loss or duplication of key regulatory genes coincides with environmental adaptation of the stomatal complex in Nymphaea colorata and Kalanchoe laxiflora
Source: Hortic Res. 2018 Aug 1;5:42. doi: 10.1038/s41438-018-0048-8 (PMC6068134; doi:10.1038/s41438-018-0048-8)

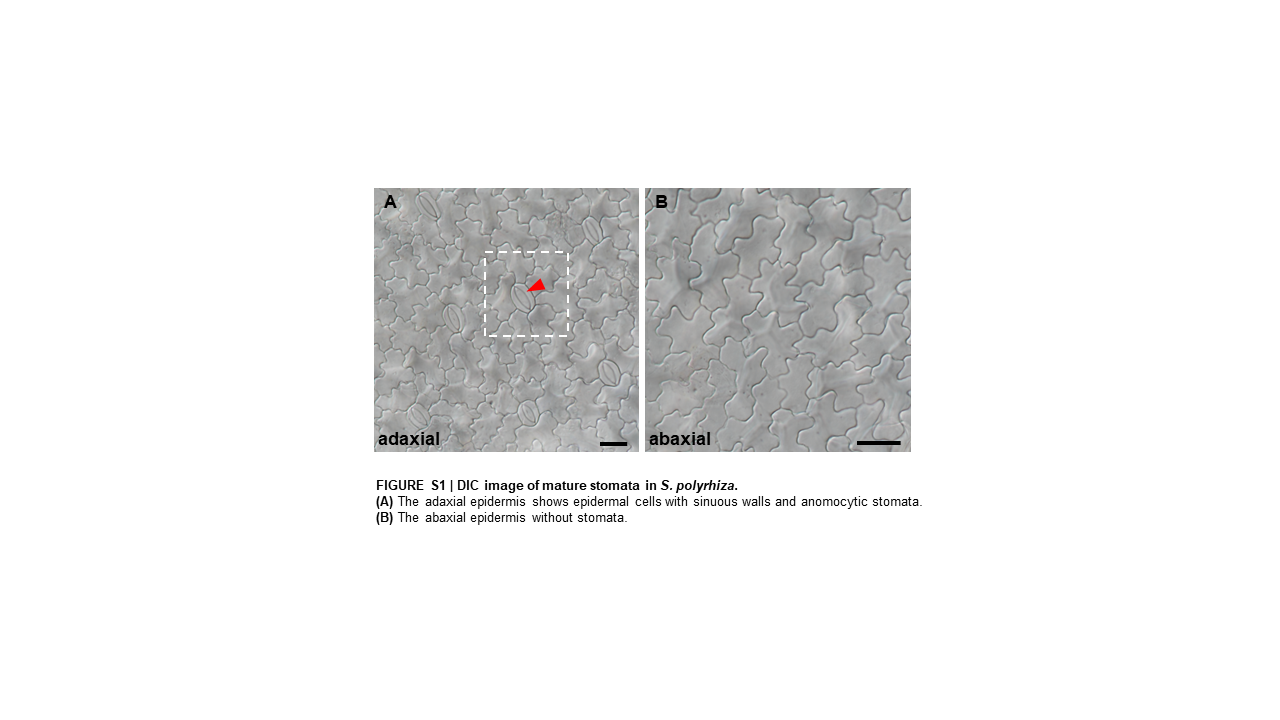

Supplement: Supplementary file 1 — Supplemental Figure 1 [file 41438_2018_48_MOESM1_ESM.tif]

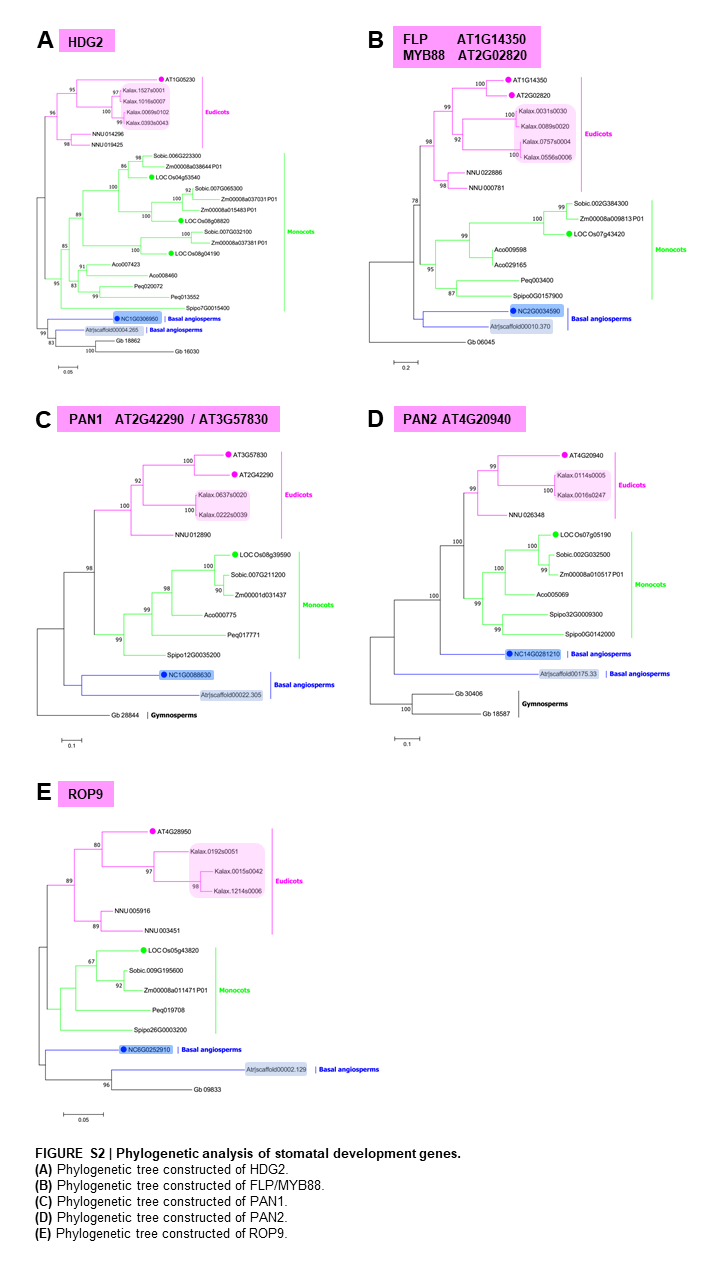

Supplement: Supplementary file 2 — Supplemental Figure 2 [file 41438_2018_48_MOESM2_ESM.tif]

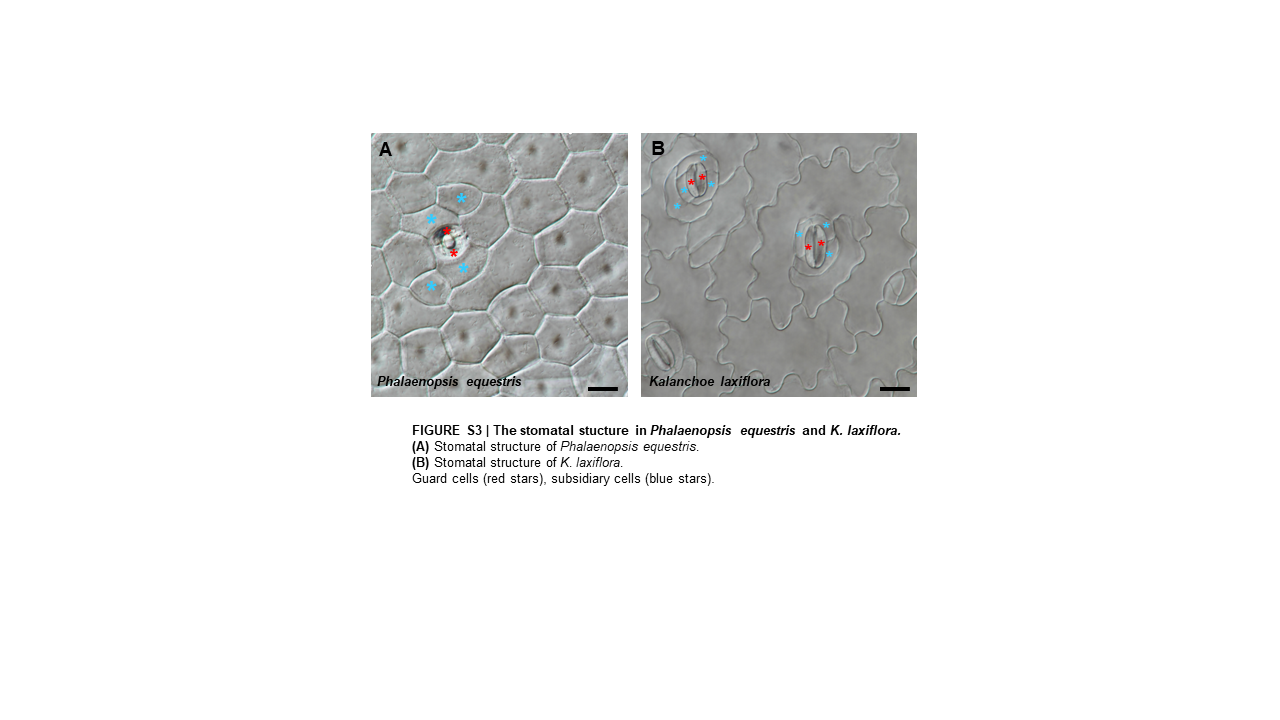

Supplement: Supplementary file 3 — Supplemental Figure 3 [file 41438_2018_48_MOESM3_ESM.tif]

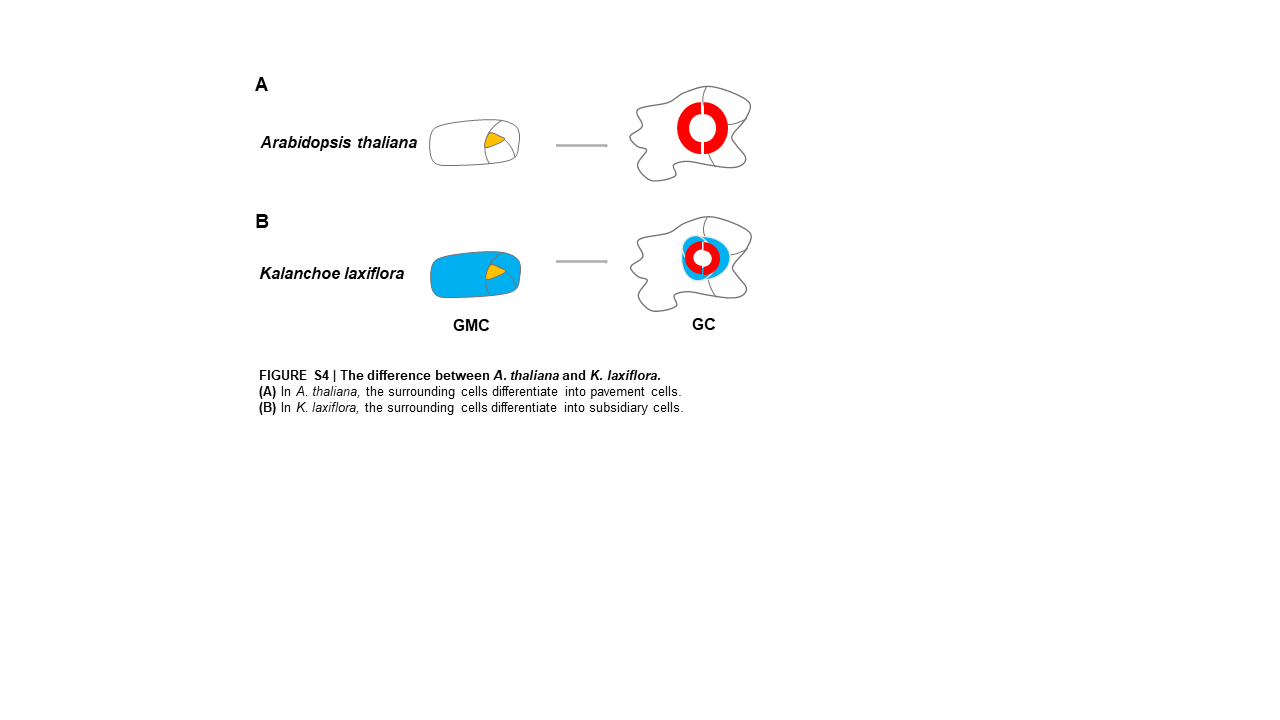

Supplement: Supplementary file 4 — Supplemental Figure 4 [file 41438_2018_48_MOESM4_ESM.tif]
